# Supplementary material for: Equity and reliability of public electric vehicle charging stations in the United States
Source: Nat Commun. 2025 Jun 18;16:5291. doi: 10.1038/s41467-025-60091-y (PMC12177045; doi:10.1038/s41467-025-60091-y)
Supplement: Supplementary file 1 — Supplementary Information [file 41467_2025_60091_MOESM1_ESM.pdf]

Supplementary Information for

Equity and reliability of public electric vehicle charging stations in the United States

Qiao Yu[1], Tristan Que[2], Lara J. Cushing[1], Gregory Pierce[3], Ke Shen[4], Mayank Kejriwal[4], Yuan Yao[1], Yifang Zhu[1*]

1. Department of Environmental Health Sciences, Fielding School of Public Health, University of California, Los Angeles, Los Angeles, CA, United States
2. Department of Computer Science, University of California, Los Angeles, Los Angeles, CA, United States
3. Luskin Center for Innovation, University of California, Los Angeles, CA, United States
4. Information Sciences Institute, University of Southern California, Marina del Rey, CA, United States

* Corresponding Author: Yifang Zhu; e-mail: yifang@ucla.edu

Table of contents

Supplementary Figure 1: Mean public electric vehicle charging stations (EVCS) numbers in 2022, comparing disadvantaged and non-disadvantaged communities

Supplementary Figure 2: Mean public electric vehicle charging station (EVCS) 3-mile radius coverage in 2022, comparing disadvantaged and non-disadvantaged communities

Supplementary Table 1: EV Charging Station 3-mile radius coverage data for the Contiguous United States and each State, comparing DAC and Non-DAC across different metrics.

Supplementary Table 2: Sensitivity analysis of disparity metrics for the Contiguous United States using 3-mile, 5-mile, and 10-mile radii for DAC vs. non-DAC comparison

Supplementary Table 3: Model performance on sentiment analysis using different models.

Supplementary Table 4: Comparison of sentiment annotations conducted by the two researchers (Interrater reliability: κ = 0.81)

Supplementary Table 5: Model performance on problem categorization using different models.

Supplementary Table 6: Comparison of problem categorization labels conducted by two researchers (Interrater reliability: κ = 0.71)

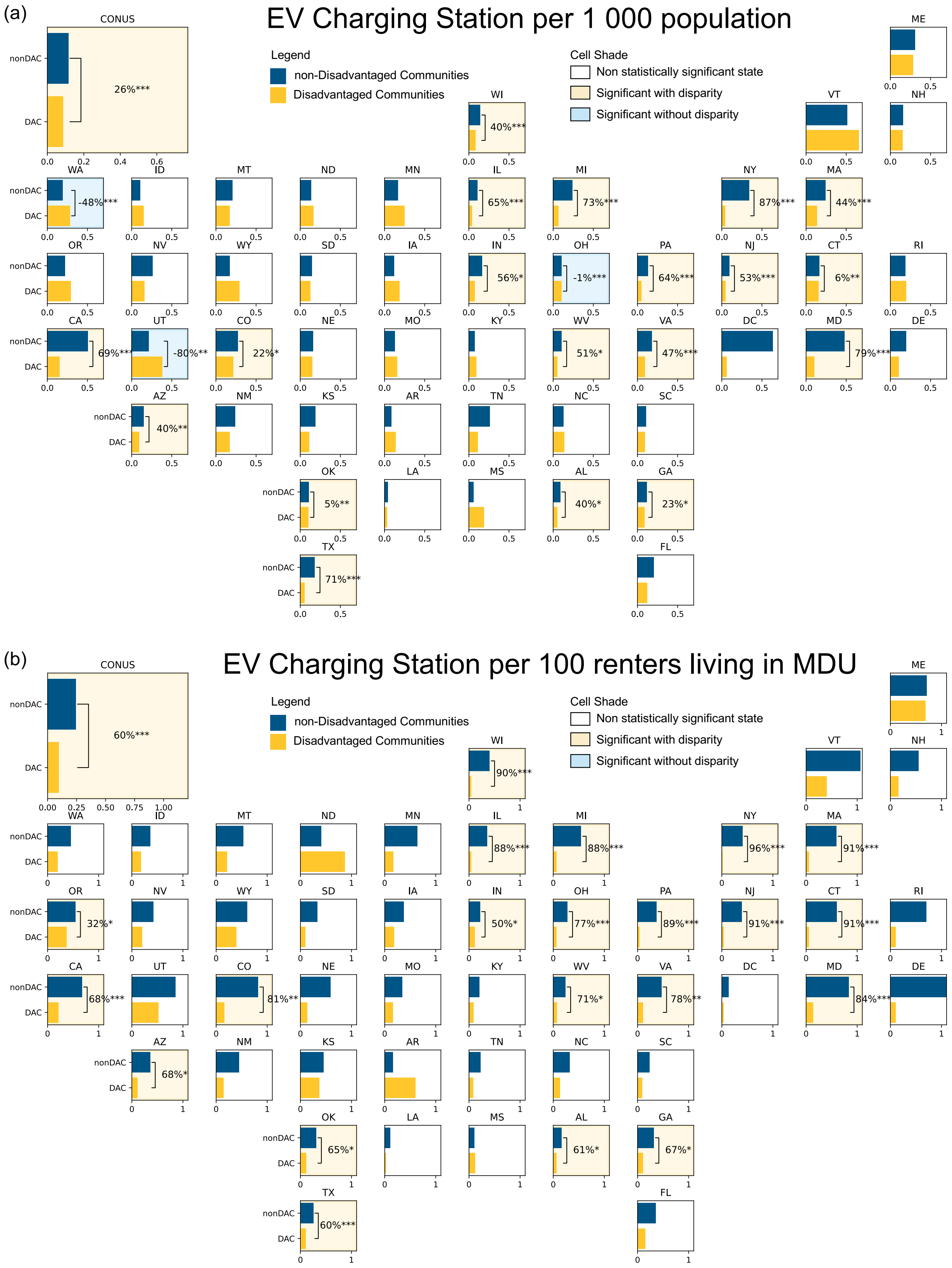
(a)
EV Charging Station per 1 000 population
CONUS
nonDAC
DAC
26%***
Legend
non-Disadvantaged Communities
Disadvantaged Communities
Cell Shade
Non statistically significant state
Significant with disparity
Significant without disparity
ME
WI
40%***
VT
NH
WA
-48%***
ID
MT
ND
MN
IL
65%***
MI
73%***
NY
87%***
MA
44%***
OR
NV
WY
SD
IA
IN
56%*
OH
-1%***
PA
64%***
NJ
53%***
CT
6%**
RI
CA
69%***
UT
-80%**
CO
22%*
NE
MO
KY
WV
51%*
VA
47%***
DC
MD
79%***
DE
AZ
40%**
NM
KS
AR
TN
NC
SC
OK
5%**
LA
MS
AL
40%*
GA
23%*
TX
71%***
FL
(b)
EV Charging Station per 100 renters living in MDU
CONUS
60%***
Legend
non-Disadvantaged Communities
Disadvantaged Communities
Cell Shade
Non statistically significant state
Significant with disparity
Significant without disparity
ME
WI
90%***
VT
NH
WA
ID
MT
ND
MN
IL
88%***
MI
88%***
NY
96%***
MA
91%***
OR
32%*
NV
WY
SD
IA
IN
50%*
OH
77%***
PA
89%***
NJ
91%***
CT
91%***
RI
CA
68%***
UT
CO
81%**
NE
MO
KY
WV
71%*
VA
78%**
DC
MD
84%***
DE
AZ
68%*
NM
KS
AR
TN
NC
SC
OK
65%*
LA
MS
AL
61%*
GA
67%*
TX
60%***
FL

Supplementary Figure 1: Mean public electric vehicle charging stations (EVCS) numbers in 2022, comparing disadvantaged and non-disadvantaged communities: (a) per 1 000 population and (b) per 100 renters living in multi-dwelling units. The yellow columns represent disadvantaged communities, while blue columns represent non-disadvantaged communities. The light yellow state cells indicate states with statistically significant disparities, the light blue state cells indicate significant but without disparity, and the white cells represent non-statistically significant states. Disparity percentage is calculated by dividing the difference between disadvantaged and non-disadvantaged communities by the value in non-disadvantaged communities. Statistical significance was evaluated using the Mann–Whitney U test (two-sided). *** $p < 0.001$, ** $p < 0.01$, * $p < 0.05$.

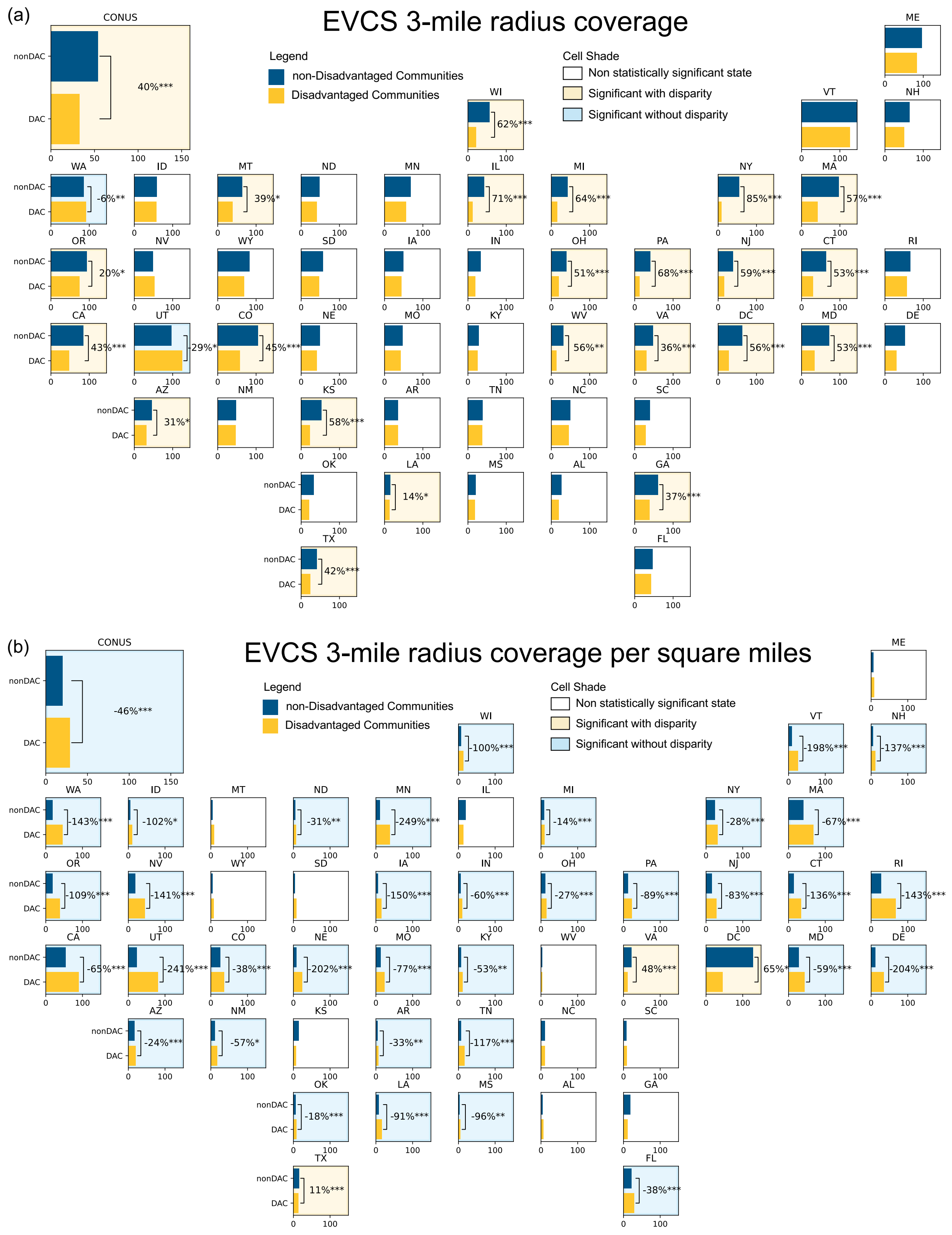
(a)
EVCS 3-mile radius coverage
Legend
non-Disadvantaged Communities
Disadvantaged Communities
Cell Shade
Non statistically significant state
Significant with disparity
Significant without disparity
CONUS
nonDAC
DAC
40%***
ME
WI
62%***
VT
NH
WA
-6%**
ID
MT
39%*
ND
MN
IL
71%***
MI
64%***
NY
85%***
MA
57%***
OR
20%*
NV
WY
SD
IA
IN
OH
51%***
PA
68%***
NJ
59%***
CT
53%***
RI
CA
43%***
UT
-29%*
CO
45%***
NE
MO
KY
WV
56%**
VA
36%***
DC
56%***
MD
53%***
DE
AZ
31%*
NM
KS
58%***
AR
TN
NC
SC
OK
LA
14%*
MS
AL
GA
37%***
TX
42%***
FL
(b)
EVCS 3-mile radius coverage per square miles
CONUS
-46%***
WI
-100%***
VT
-198%***
NH
-137%***
WA
-143%***
ID
-102%*
MT
ND
-31%**
MN
-249%***
IL
MI
-14%***
NY
-28%***
MA
-67%***
OR
-109%***
NV
-141%***
WY
SD
IA
-150%***
IN
-60%***
OH
-27%***
PA
-89%***
NJ
-83%***
CT
-136%***
RI
-143%***
CA
-65%***
UT
-241%***
CO
-38%***
NE
-202%***
MO
-77%***
KY
-53%**
WV
VA
48%***
DC
65%*
MD
-59%***
DE
-204%***
AZ
-24%***
NM
-57%*
KS
AR
-33%**
TN
-117%***
NC
SC
OK
-18%***
LA
-91%***
MS
-96%**
AL
GA
TX
11%***
FL
-38%***

Supplementary Figure 2: Mean public electric vehicle charging station (EVCS) 3-mile radius coverage in 2022, comparing disadvantaged and non-disadvantaged communities: (a) total coverage, and (b) coverage per square mile. The red columns represent disadvantaged communities, while green columns represent non-disadvantaged communities. The light yellow state cells indicate states with statistically significant disparities, the light blue state cells indicate significant but without disparity, and the white cells represent non-statistically significant states. Disparity percentage is calculated by dividing the difference between disadvantaged and non-disadvantaged communities by the value in non-disadvantaged communities. Statistical significance was evaluated using the Mann–Whitney U test (two-sided). *** $p < 0.001$, ** $p < 0.01$, * $p < 0.05$.

Supplementary Table 1: EV Charging Station 3-mile radius coverage data for the Contiguous United States and each State, comparing DAC and Non-DAC across different metrics.

*Only values with an asterisk sign has statistically significant results between DAC and non-DAC ($p<0.05$). Statistical significance was evaluated using the Mann–Whitney U test (two-sided).

State	EVCS 3-mile coverage		EVCS 3-mile coverage									
			per square miles		per 1 000 population		per population density		per 1 000 renters		per 100 renters living in MDU	
	DAC	non-DAC	DAC	non-DAC	DAC	non-DAC	DAC	non-DAC	DAC	non-DAC	DAC	non-DAC
US	32.82*	54.28*	29.07*	19.89*	9.96*	27.94*	0.41*	2.74*	22.27*	89.67*	13.94*	51.74*
AL	19.99	26.50	6.37	4.77	7.34	6.57	0.22	0.24	20.26	32.71	17.17	19.43
AR	34.91	35.49	6.77*	5.11*	12.43*	8.13*	0.58	0.54	28.60	32.66	57.59	22.33
AZ	31.20*	45.52*	20.59*	16.61*	8.48	23.74	0.62*	4.33*	19.06*	69.62*	17.18	48.50
CA	48.01*	84.63*	89.33*	54.10*	15.51*	63.57*	0.09*	3.49*	31.54*	100.30*	22.78*	89.47*
CO	57.88*	104.70*	35.76*	25.83*	15.23*	32.61*	3.54*	10.22*	35.99*	195.31*	21.83*	101.46*
CT	29.89*	63.73*	33.93*	14.38*	9.69*	15.66*	0.01*	0.26*	15.08*	132.78*	4.07*	79.75*
DC	27.32*	62.33*	45.50*	128.36*	8.67*	207.65*	0.01*	1.44*	12.65*	227.94*	3.96*	44.92*
DE	30.52	52.71	34.32*	11.31*	8.95	14.23	0.03*	0.26*	19.91*	215.39*	15.88	76.16
FL	42.73	46.38	29.27*	21.22*	10.72	14.44	0.10*	3.24*	22.64*	81.21*	16.18*	37.84*
GA	38.26*	60.45*	10.63	18.55	9.50	11.12	0.26*	0.29*	19.00*	51.65*	11.43*	36.48*
IA	44.22	49.10	14.90*	5.96*	13.12*	11.88*	0.14	1.58	36.48	78.26	16.43	46.11
ID	57.96	58.90	10.84*	5.36*	11.38	11.34	2.04	13.16	28.96	59.71	14.75	50.10
IL	12.61*	42.78*	13.01	20.12	3.58*	10.29*	0.02*	0.36*	8.62*	70.91*	3.65*	41.18*
IN	19.35	33.62	9.85*	6.14*	6.73	11.46	0.04*	0.28*	14.88*	60.92*	11.12	26.86
KS	22.46*	53.02*	6.88	14.23	7.74*	18.44*	0.11*	1.69*	18.14*	116.44*	26.75*	58.46*
KY	25.90	28.77	11.19*	7.32*	8.25	7.22	0.22	0.27	19.66	40.09	11.65	21.18
LA	13.07*	15.18*	15.96*	8.37*	5.71*	5.79*	0.03	0.14	11.76*	15.63*	19.27*	8.70*
MA	42.13*	97.61*	67.64*	40.57*	10.71*	27.73*	0.01*	0.35*	17.68*	177.38*	4.72*	70.69*
MD	34.15*	72.05*	43.97*	27.63*	10.84*	70.54*	0.03*	0.80*	23.10*	149.42*	18.44*	93.37*
ME	83.60	96.87	8.40	6.23	24.35	26.85	11.62	2.46	135.42*	166.60*	69.25*	78.14*
MI	15.49*	42.90*	10.44*	9.19*	6.55*	24.53*	0.04*	1.00*	15.60*	116.21*	9.55*	60.87*
MN	56.69	68.11	38.35*	10.98*	23.06	16.64	1.08*	4.15*	47.14*	122.53*	15.50*	78.73*
MO	41.79	46.83	23.62*	13.34*	18.03	18.50	0.23*	0.87*	50.09*	66.66*	21.65	43.02
MS	18.30	21.00	5.43*	2.76*	7.40*	5.15*	0.18	0.28	15.74	19.39	11.80	9.53
MT	38.66*	63.39*	9.12	4.51	11.97	23.06	9.12*	43.84*	34.23*	81.20*	21.56*	56.08*
NC	46.09	49.39	11.30	11.15	12.90*	12.30*	0.32	0.28	28.45*	58.03*	16.38	35.57
ND	41.35	48.37	7.21*	5.51*	16.00	13.39	11.31	14.56	50.64	77.97	75.74	50.74
NE	40.76	49.21	24.56*	8.12*	12.81	17.47	0.11*	6.15*	30.96	89.55	16.37	60.74
NH	50.75	64.76	11.90*	5.03*	13.66	15.13	2.71	1.93	52.11	96.41	16.09	51.50
NJ	15.59*	38.29*	28.85*	15.78*	4.64*	9.45*	0.02*	0.08*	9.75*	94.67*	3.61*	44.09*

NM	46.88	47.66	17.36*	11.02*	12.48	15.07	6.08	11.76	32.41	71.42	14.06	53.80
NV	53.03	48.88	44.87*	18.65	15.45*	23.96*	0.09	21.16	26.53	65.03	8.55	49.16
NY	8.44*	54.50*	32.00*	24.98*	3.62*	73.57*	0.01*	1.20*	5.08*	118.46*	1.43*	49.27*
OH	19.46*	39.41*	14.71*	11.62*	8.18	9.73	0.03*	0.21*	15.82*	63.85*	7.85*	35.38*
OK	20.63	32.90	7.99*	6.76*	8.67	21.80	0.24*	1.14*	25.64*	58.10*	19.72	41.56
OR	74.89*	93.71*	38.36*	18.38*	21.20*	21.44*	0.33*	14.07*	48.58*	94.85*	31.00*	68.45*
PA	13.01*	40.50*	22.76*	12.06*	4.26*	13.12*	0.01*	0.32*	9.13*	90.37*	5.24*	43.37*
RI	57.69	66.04	67.44*	27.79*	14.93	15.39	0.03*	0.12*	26.24*	89.84*	7.72*	52.83*
SC	28.73	39.31	8.74	7.38	8.36	9.52	0.17	0.24	18.68*	46.25*	13.31	24.82
SD	47.12	57.43	8.16	4.19	11.26	14.50	2.39*	10.19*	33.77*	78.78*	12.59*	34.69*
TN	37.80	38.47	16.45*	7.57*	11.85*	25.01*	0.11	0.61	22.98	58.79	11.35	30.49
TX	23.68*	40.80*	13.74*	15.38*	5.30*	16.70*	0.03*	1.96*	12.22*	75.40*	12.67*	32.29*
UT	124.53*	96.74*	81.43*	23.87*	32.24*	22.50*	2.56	25.56	76.67	123.40	45.33*	110.47*
VA	30.39*	47.71*	11.14*	21.27*	8.11*	72.94*	0.26*	0.66*	20.30*	127.93*	9.71*	61.68*
VT	126.34	160.57	25.53*	8.56*	39.21	52.31	1.43*	4.98*	116.31*	298.74*	30.67*	118.86*
WA	91.38*	86.17*	45.75*	18.86*	21.43*	18.69*	0.24*	7.22*	45.79*	94.23*	16.77*	69.84*
WI	21.39*	56.55*	13.83*	6.91*	6.53*	13.27*	0.08*	1.79*	13.30*	84.81*	3.75*	49.13*
WV	13.95*	31.98*	3.15	3.22	5.43	9.24	0.29*	1.08*	18.21*	48.73*	8.82*	26.08*
WY	68.41	82.64	8.09	4.66	18.48	19.16	12.75	64.88	59.23	97.33	32.37	106.73

Supplementary Table 2: Sensitivity analysis of disparity metrics for the Contiguous United States using 3-mile, 5-mile, and 10-mile radii for DAC vs. non-DAC comparison

EVCS Kernel Density	DAC classification	3-mile radius	Disparity %	5-mile radius	Disparity %	10-mile radius	Disparity %
per square miles	DAC	29.1	-46.1%	279	-55.2%	963	-62.9%
	non-DAC	19.9		180		591	
per 1 000 people	DAC	9.96	64.4%	87.8	69.4%	304	72.8%
	non-DAC	27.9		287		1120	
adjusted for population density	DAC	0.41	85.0%	3.11	88.1%	14.4	88.4%
	non-DAC	2.73		26.1		124	
per 1 000 renters	DAC	22.3	75.2%	195	76.8%	723	81.6%
	non-DAC	89.6		841		3920	
per 100 renters live in MDU	DAC	13.9	73.1%	134	75.0%	570	78.2%
	non-DAC	51.7		533		2620	

As the KDE radius expands from 3 to 5 and 10 miles, **EVCS density per square mile** increases more in DAC areas. This is due to DAC areas' higher density clustering, which benefits from the larger radius. However, when adjusting for population or population density, **per capita EVCS coverage in DACs appears diluted**, as the higher population density in DACs requires proportionally more EVCS density to meet demand. Thus, even as spatial coverage improves, DAC areas continue to face an EVCS coverage gap, highlighting a persistent disparity in population-adjusted EVCS coverage.

Supplementary Table 3: Model performance on sentiment analysis using different models.

Model	Accuracy (%) (s.d.)	Precision	Recall	F1 score
CNN (Asensio et al., 2020)	84.7 (0.8)	0.86	0.86	0.86
GPT 3.5	86.2 (0.02)	0.95	0.81	0.88
GPT 4	87.1 (0.01)	0.96	0.82	0.88

GPT 3.5 model: gpt-3.5-turbo-0125
GPT 4 model: gpt-4-0125-preview

Supplementary Table 4: Comparison of sentiment annotations conducted by the two researchers (Interrater reliability: κ = 0.81)

	Researcher 1	Researcher 2
Positive Annotation	1 885 (59.1%)	2 034 (63.8%)
Negative Annotation	1 303 (40.9%)	1 154 (36.2%)
Total	3 188 (100.0%)	3 188 (100.0%)

Supplementary Table 5: Model performance on problem categorization using different models.

Model	Accuracy (%) (s.d.)	Precision	Recall	F1 score
GPT 3.5	76.5 (0.19)	0.83	0.76	0.79
GPT 4	82.4 (0.08)	0.87	0.82	0.84

GPT 3.5 model: gpt-3.5-turbo-0125
GPT 4 model: gpt-4-0125-preview

Supplementary Table 6: Comparison of problem categorization labels conducted by two researchers (Interrater reliability: κ = 0.71)

Problem Category	Researcher 1	Researcher 2
Hardware and Technical Issues	571 (52.8%)	556 (51.4%)
Software and Digital Infrastructure	34 (3.1%)	44 (4.1%)
User Experience	137 (12.7%)	143 (13.2%)
Restrictions and Accessibility	265 (24.5%)	286 (26.5%)
Cost Concerns	28 (2.6%)	20 (1.9%)
Incomprehensible comments	46 (4.3%)	32 (3.0%)
Total	1 081 (100.0%)	1 081 (100.0%)
