# Supplementary material for: Equity and reliability of public electric vehicle charging stations in the United States
Source: Nat Commun. 2025 Jun 18;16:5291. doi: 10.1038/s41467-025-60091-y (PMC12177045; doi:10.1038/s41467-025-60091-y)
Supplement: Supplementary file 2 — Reporting Summary [file 41467_2025_60091_MOESM2_ESM.pdf]

nature portfolio

Corresponding author(s): Yifang Zhu

Last updated by author(s): Apr 15, 2025

Reporting Summary

Nature Portfolio wishes to improve the reproducibility of the work that we publish. This form provides structure for consistency and transparency in reporting. For further information on Nature Portfolio policies, see our Editorial Policies and the Editorial Policy Checklist.

Statistics

For all statistical analyses, confirm that the following items are present in the figure legend, table legend, main text, or Methods section.

n/a	Confirmed	
☐	☒	The exact sample size (*n*) for each experimental group/condition, given as a discrete number and unit of measurement
☒	☐	A statement on whether measurements were taken from distinct samples or whether the same sample was measured repeatedly
☐	☒	The statistical test(s) used AND whether they are one- or two-sided<br>*Only common tests should be described solely by name; describe more complex techniques in the Methods section.*
☒	☐	A description of all covariates tested
☒	☐	A description of any assumptions or corrections, such as tests of normality and adjustment for multiple comparisons
☐	☒	A full description of the statistical parameters including central tendency (e.g. means) or other basic estimates (e.g. regression coefficient) AND variation (e.g. standard deviation) or associated estimates of uncertainty (e.g. confidence intervals)
☐	☒	For null hypothesis testing, the test statistic (e.g. *F*, *t*, *r*) with confidence intervals, effect sizes, degrees of freedom and *P* value noted<br>*Give P values as exact values whenever suitable.*
☒	☐	For Bayesian analysis, information on the choice of priors and Markov chain Monte Carlo settings
☒	☐	For hierarchical and complex designs, identification of the appropriate level for tests and full reporting of outcomes
☒	☐	Estimates of effect sizes (e.g. Cohen's *d*, Pearson's *r*), indicating how they were calculated

Our web collection on statistics for biologists contains articles on many of the points above.

Software and code

Policy information about availability of computer code

Data collection	AFDC EVCS location data: a dataset provided by the U.S. Department of Energy, offers publicly available information on electric vehicle charging station locations across the United States.<br>DOE Disadvantaged Communities Reporter: a tool provided by the U.S. Department of Energy, identifies and maps disadvantaged communities to support energy justice initiatives.<br>US Census data: 2010 ACS Table B08013 and Subject Table S2504 provide information on commuting characteristics and housing unit features across the United States.<br>EVCS review data: user-generated content from an online review platform providing information on EVCS locations and related reviews.
Data analysis	Python 3.12.2: an open-source versatile high-level programming language used in web development, data analysis, and AI.<br>Pandas 2.2.1: a Python package for flexible data manipulation and analysis.<br>NumPy 1.26.4: a Python package supporting large, multi-dimensional arrays and matrices.<br>SciPy 1.12.0: a Python package for scientific computing and technical computing.<br>GeoPandas 0.14.3: a Python package for easier handling of geospatial data.

For manuscripts utilizing custom algorithms or software that are central to the research but not yet described in published literature, software must be made available to editors and reviewers. We strongly encourage code deposition in a community repository (e.g. GitHub). See the Nature Portfolio guidelines for submitting code & software for further information.

Data

Policy information about availability of data

All manuscripts must include a data availability statement. This statement should provide the following information, where applicable:

- Accession codes, unique identifiers, or web links for publicly available datasets
- A description of any restrictions on data availability
- For clinical datasets or third party data, please ensure that the statement adheres to our policy

The EVCS location data from the U.S. DOE are publicly available from the AFDC website (https://afdc.energy.gov/stations#/find/nearest). Environmental justice data were downloaded from DOE Disadvantaged Communities Reporter (https://energyjustice.egs.anl.gov). However, due to government changes, these data are no longer available as of 2025. A backup of the dataset has been uploaded to the Figshare repository at: https://figshare.com/s/1cca6ebb81aae425e5ba. Additional Census data are available from the United States Census Bureau (https://data.census.gov). Data from the user-generated platform contain identifiable information and cannot be posted publicly due to privacy restrictions. A de-identified version is available from the corresponding author upon request. Source data are provided with this paper. All codes and prompts for sentiment analysis and problem categorization have been deposited in the Figshare repository at: https://figshare.com/s/209509bbc5067c936ce3

Research involving human participants, their data, or biological material

Policy information about studies with human participants or human data. See also policy information about sex, gender (identity/presentation), and sexual orientation and race, ethnicity and racism.

Reporting on sex and gender	This information has not been collected.
Reporting on race, ethnicity, or other socially relevant groupings	This information has not been collected.
Population characteristics	Not applicable.
Recruitment	Not applicable.
Ethics oversight	Not applicable.

Note that full information on the approval of the study protocol must also be provided in the manuscript.

Field-specific reporting

Please select the one below that is the best fit for your research. If you are not sure, read the appropriate sections before making your selection.

☐ Life sciences ☐ Behavioural & social sciences ☒ Ecological, evolutionary & environmental sciences

For a reference copy of the document with all sections, see nature.com/documents/nr-reporting-summary-flat.pdf

Ecological, evolutionary & environmental sciences study design

All studies must disclose on these points even when the disclosure is negative.

Study description	Our study analyzes the distribution of electric vehicle charging station locations in the continental United States in 2022, as well as their reliability and user experience using review data from an online platform. This study is a secondary data analysis that does not involve any human interviews, laboratory experimentation, or field data collection. The data used in this research were collected from publicly accessible databases or online platforms.
Research sample	The study sample includes publicly accessible EVCS stations within the continental United States, along with user-generated comments and reviews from individuals who have used and evaluated these stations. The analysis utilized existing data from two sources: (1) AFDC EVCS location data, a dataset provided by the U.S. Department of Energy offering publicly available information on electric vehicle charging station locations across the United States; and (2) EVCS review data, user-generated content from an online review platform providing information on EVCS locations and related reviews. Demographic information about the users who posted reviews is not available, as this data was not collected by the platform.
Sampling strategy	For user sentiment analysis validation, we employed a random sampling procedure to select 3,188 non-empty comments from the entire dataset of user reviews. We used the pandas.DataFrame.sample function in Python. We believe this sample size is sufficient, as it aligns with sample sizes used in previous studies validating sentiment analysis methods.
Data collection	The data sources for this study include the AFDC EVCS location data, the US DOE Disadvantaged Communities Reporter, the US Census data (2010 ACS Table B08013 and Subject Table S2504), and EVCS review data. These datasets were collected by various U.S. federal agencies and an online EV charging station review platform. All data were downloaded from publicly accessible databases or the respective platforms for analysis.

Timing and spatial scale	We did not conduct primary data collection. However, all of our data were downloaded in October 2022 from the aforementioned data sources for the continental United States.
Data exclusions	We excluded EV charging station reviews that were empty or contained only spaces during the data cleaning process for sentiment analysis.
Reproducibility	The study ensures high reproducibility by utilizing publicly accessible raw data and open-source tools for data analysis.
Randomization	As this study does not involve primary data collection or participants, randomization and covariate control are not applicable. For sentiment analysis validation, we used the pandas.DataFrame.sample function in Python to randomly select samples for validation.
Blinding	For sentiment analysis validation, we trained human annotators to establish a consensus on the criteria for categorizing reviews as positive or negative. Annotators were informed that the task involved labeling EVCS reviews but were not provided with additional context or hypotheses about the study to minimize potential bias. They then independently labeled a randomly selected subset of comments to test inter-rater reliability and assess the quality of the data for validation purposes. A similar approach was employed for labeling negative comment categories, with two annotators performing the task independently.

Did the study involve field work? ☐ Yes ☒ No

Reporting for specific materials, systems and methods

We require information from authors about some types of materials, experimental systems and methods used in many studies. Here, indicate whether each material, system or method listed is relevant to your study. If you are not sure if a list item applies to your research, read the appropriate section before selecting a response.

Materials & experimental systems

n/a	Involved in the study
☒	☐ Antibodies
☒	☐ Eukaryotic cell lines
☒	☐ Palaeontology and archaeology
☒	☐ Animals and other organisms
☒	☐ Clinical data
☒	☐ Dual use research of concern
☒	☐ Plants

Methods

n/a	Involved in the study
☒	☐ ChIP-seq
☒	☐ Flow cytometry
☒	☐ MRI-based neuroimaging

Plants

Seed stocks	*Report on the source of all seed stocks or other plant material used. If applicable, state the seed stock centre and catalogue number. If plant specimens were collected from the field, describe the collection location, date and sampling procedures.*
Novel plant genotypes	*Describe the methods by which all novel plant genotypes were produced. This includes those generated by transgenic approaches, gene editing, chemical/radiation-based mutagenesis and hybridization. For transgenic lines, describe the transformation method, the number of independent lines analyzed and the generation upon which experiments were performed. For gene-edited lines, describe the editor used, the endogenous sequence targeted for editing, the targeting guide RNA sequence (if applicable) and how the editor was applied.*
Authentication	*Describe any authentication procedures for each seed stock used or novel genotype generated. Describe any experiments used to assess the effect of a mutation and, where applicable, how potential secondary effects (e.g. second site T-DNA insertions, mosiacism, off-target gene editing) were examined.*
